# Daily Supplementation with *Bifidobacterium longum KACC91563* Alleviates Allergic Contact Dermatitis in an Animal Model

**DOI:** 10.3390/foods13142190

**Published:** 2024-07-11

**Authors:** Van-Ba Hoa, So-Hyun Park, Do-Hyun Ha, Je-Hee Son, Kil-Ho Lee, Won-Seo Park, Ja-Yeon Yoo, In-Seon Bae, Hyoun-Wook Kim, Han-Byul Kang, Sang-Myeong Lee, Jun-Sang Ham

**Affiliations:** 1 Animal Products Utilization Division, National Institute of Animal Science, Rural Development Administration (RDA), Wanju-gun 55365, Republic of Korea; 2 College of Veterinary Medicine, Chungbuk National University, Cheongju-si 28644, Republic of Korea; 3 Division of Biotechnology, Jeonbuk National University, Iksan 54596, Republic of Korea

**Keywords:** allergic disease, probiotic *B. longum*, daily supplementation

## Abstract

Allergic contact dermatitis (ACD) is the most common chronic inflammatory skin disease (or immune-mediated disease), causing disruption to our psychological condition and life quality. In this study, the therapeutic properties of probiotic *Bifidobacterium longum* (*B. longum*) was investigated by using an ACD-induced animal model. For ACD induction, BALB/c mice ear and dorsal skin were sensitized with 240 µL of 1% (*w*/*v*) 2,4-dinitrochlorobenzene (DNCB) twice (3-day intervals). After a week of the first induction, the mice were re-sensitized by painting on their dorsal skin and ear with 0.4% (*w*/*v*) DNCB for a further three times (once per week). Before the ACD induction of 2 weeks and throughout the trial period, the BALB/c mice were supplemented daily with 1 mL of 1.0 × 10^9^ CFU or 5.0 × 10^9^ CFU *B. longum* using an intragastric gavage method. The ACD-induced mice without *B. longum* supplementation were used as a control. Results show that *B. longum* supplementation significantly alleviated ACD symptoms (e.g., ear swelling, epidermal damage) and immune response (e.g., reduced immune cell recruitment, serum IgE level, and cytokine production). The therapeutic efficiency of *B. longum* increased as the supplementation dose increased. Thus, daily supplementation with 5.0 × 10^9^ CFU probiotic *B. longum* could be an effective method for the prevention and treatment of ACD.

## 1. Introduction

Nowadays, consumers are aware of the effects of the foods that they consume on their health. A general trend that has been observed is that there is an increasing consumer demand for foods that lead to enhanced health conditions, such as better skin, enhanced memory, and boosted immune system function. This is because there is a causality relationship between dietary patterns and chronic diseases [1,2]. Researchers have reported that healthy dietary patterns are associated with a decreased risk of chronic diseases [3,4]. Therefore, the consumption of healthy foods, such as omega-3 fatty acids, antioxidants, vitamins—rich ones, and probiotics, is considered the most cost-effective way to prevent chronic diseases [5,6,7,8].

Allergic contact dermatitis (ACD) is the most common chronic inflammatory skin disease occurring in people of all ages [9]. ACD severely affects patient’s psychological condition and life quality since it causes local skin rash, itchiness, redness, swelling, lesions and necrosis [10]. ACD, as an immune-mediated disease, is caused by the direct contact of skin with exogenous reactive substances (called hapten or allergen) to which we have previously been sensitized [10]. Until now, it has been estimated that there are about 3000 chemicals that cause ACD [11]. The pathophysiology of ACD mainly consists of sensitization (induction) and elicitation phases, which both involve innate and adaptive immune responses. In the initial phase, an allergen penetrates into the outer layer of skin, which activates the proliferation and differentiation of lymphocytes (effector T cells); the second phase occurs when the sensitized individuals are re-exposed to the same allergen, which is subsequently presented to the memory T-cells and triggers an adaptive immune response reaction [12]. As a result of immune responses, a large number of cytokines and chemokines are secreted in the skin by the T cells, keratinocytes, mast cells, etc. These secreted cytokines, such as interferon-gamma (IFN-γ), tumor necrosis factor alpha (TNF), and interleukin (IL)-4, IL-5, IL-12, IL-13, IL-17, IL-22, etc., act as signals for the communication of immune cells and the regulation of the immune responses [13].

It is well recognized that gut microbiota composition largely affects gastrointestinal tract health since it is associated with the absorption of nutrients (e.g., minerals and vitamins) and the synthesis of essential substances (e.g., short-chain fatty acids) [14]. Any alteration of the intestinal microbiota composition usually results in an immune response disorder and the development of skin diseases [15]. In recent decades, probiotics have been proven to be an immunomodulator and are widely used as an adjuvant therapy for ACD cases [8,16,17]. The mechanisms responsible for this phenomenon are in the probiotics with live microorganisms, which beneficially affect the gut microbiota and intestinal barrier function, resulting in a reduced inflammatory reaction in allergy diseases [8,18]. Human studies have reported that daily supplementation of *Lactiplantibacillus plantarum* (*L. plantarum* LM1004) for 8 weeks shows a therapeutic effect against dermatitis in patients [19]. Prakoeswa et al. [20] reported a decrease in clinical symptoms, levels, serum immunoglobulin E (IgE), interleukin production, and adaptive immune response in patients with ACD after being supplemented with probiotic *L*. *plantarum* IS-10506. Furthermore, an animal model has widely been used in studies with probiotic microorganisms on skin diseases. The oral administration of *Lacticaseibacillus casei* (*L. casei* DN-114-001) has been shown to reduce the contact hypersensitivity response (e.g., reduced IFN-γ production) induced by allergens such as 1-fluoro-2,4-dinitrobenzene (DNFB) in mice [21,22]. The proliferation of supplemented probiotic microorganisms in the gastrointestinal tract has been proposed to be the main reason responsible for the reduction of responses in mice with ACD [22]. Hence, the supplementation of dietary probiotics seems to emerge as a cost-effective alternative for the prevention and alleviation of ACD through immune response modulation and anti-inflammatory response.

Among the gut microbiota composition, bifidobacteria are Gram-positive and non-spore-forming bacteria commonly found in the human intestinal tract, with different ratios depending on age (e.g., it accounts for 90% of the total bacteria in breast-fed infant’s feces and only about 3% to 5% in adult’s feces [23]). Bifidobacteria have been found to exert wide range of biological activities, such as anti-carcinogenesis, serum cholesterol lowering, immune system enhancement and antimicrobial activity, and vitamin B synthesis [24]. Until now, several *Bifidobacterium* spp. (e.g., *B. longum* 51A) have been studied and used as probiotic microorganisms [22,25]. In our previous study, a strain of *Bifidobacterium*, isolated from fecal samples of healthy Korean neonates was sequenced and identified as *B. longum* [23]. The *B. longum* was registered at the Korean Agriculture Cultural Collection (KACC) as KACC91563 and, in GenBank, with an accession number CBG0412011-1. The *B. longum* KACC91563 has been characterized for its probiotic properties and used as a starter culture to enhance the fermenting process in food products, such as salami [26] and cheese [27]. Recently, it has also been proven to be an immunomodulator, as it inhibits cytokine production in T helper type 2 (Th2) cells and reduces immunoglobulin E in mouse splenocytes and macrophages [28]. 

Furthermore, with the health benefits, probiotic bacteria have been used in the manufacture of food products (e.g., dairy products and foods with encapsulated probiotics) and dietary supplements [29]. Researchers have proposed that direct intake of foods containing probiotics has the same treatment effect on ACD [30]. Although *B. longum* KACC91563 has been proven to exert the probiotic and immunomodulatory properties mentioned above, it still remains unknown whether this bacterial strain exhibits a therapeutic effect on ACD symptoms. Thus, the motivation of this study was to assess the therapeutic effect of *B. longum* KACC91563 on the ACD through an animal model. Our study provides an important scientific basis for determining the dose of *B. longum* KACC91563 that may be incorporated into food products or dietary supplements for effective alleviation of ACD.

## 2. Materials and Methods

### 2.1. Reagents

2,4-Dinitrochlorobenzene (DNCB), olive oil, chloroform, and other reagents were purchased from Sigma-Aldrich (St. Louis, MO, USA). Mouse IgE ELISA kit, red blood cell lysis buffer, PMA/ION, brefeldin A, T-per lysis buffer, protease and phosphatase inhibitors, antibodies, and substrates for Western blot were purchased from Thermofisher Scientific (Carlsbad, CA, USA). Anaeropack gas generator and Mitsubishi anaero pack rectangular jar 2.5L were purchased from Thermo Scientific Inc. (Waltham, MA, USA). All media (e.g., MRS broth) were purchased from BD Biosciences (San Jose, CA, USA). Female BALB/c mice at 5-week-old and standard diet were purchased from Samtako, Inc. (Osan, Republic of Korea).

### 2.2. Animals

All procedures involved in the use of animals are followed by the Guide for the Care and Use of Laboratory Animals by the US National Institutes of Health [31]. In this study, the animal experimentation protocol was approved by the Institutional Animal Care and Use Committee of Chonbuk National University (Approved No: CBNU-2019-00128). The mice were housed in individually ventilated cages (6 mice each), bedded with Corn cob, and given free access to a standard diet and water in a room with controlled environment: 12 h dark–light cycle, temperature of 22–25 °C, and relative humidity of 45–55%.

### 2.3. Preparation of Bifidobacterium longum

*Bifidobacterium longum* (*B. longum* KACC91563), isolated and characterized in our previous study [23], was used in this study. The stock was firstly inoculated on MRS agar plates for 3 days under anaerobic conditions using an anaerobic gas generator and a Mitsubishi an aero pack rectangular jar 2.5L. Thereafter, the colonies were picked up from the plates and cultured in the MRS for 3 days under the same conditions. The number of cells was determined by plating serial dilutions on the same agar medium plates and conditions. Thereafter, cells were collected and washed with phosphate buffer saline (PBS pH 7.4) and freeze-dried. Finally, cells were diluted to $1\times 10^{9}$ colony-forming units (CFU)/mL and $5\times 10^{9}$ CFU/mL with saline solution prior to use. 

### 2.4. ACD Model Induction and Treatment with B. longum

Prior to the trial, the animals were acclimated for 1 week, and then they were randomly assigned into different groups (6 mice each) as follows:

ACD-induced mice with *B. longum* treatment: Two weeks before ACD induction and throughout the experimental period, the mice were administered 1.0 mL of saline solution containing $1\times 10^{9}$ CFU or $5\times 10^{9}$ CFU *B. longum* daily by intragastric gavage using a stainless-steel needle with a rounded ball tip. The needle was gently inserted into the esophagus and advanced into the stomach, and the *B. longum* was slowly administered. Care was taken to avoid causing any injury to the animals. Two weeks after the *B. longum* administration, the mice’s back skin was shaved for ACD induction. Briefly, the ear and dorsal skin areas were sensitized with 240 µL of 1% (*w*/*v*) DNCB in acetone and olive oil (3:1, *v*/*v*) mixture 2 times (every 3 days). A week after the first induction, the dorsal skin and ears were re-sensitized by painting with 200 µL and 20 µL (each ear) of 0.4% (*w*/*v*) DNCB mixture, respectively. This re-sensitizing step was repeated with 0.4% (*w*/*v*) DNCB mixture for a further 3 times (once per week).

ACD-induced mice without *B. longum* treatment: To examine whether the probiotic *B. longum* exerts the therapeutic properties for the ACD, a group of ACD-induced mice (the ACD induction was carried out using the same procedure as described above) was administered 1.0 mL saline solution daily using the same method (intragastric gavage) as described above. 

For the control group: The mice in this group were not induced by ACD treatment and did not receive *B. longum* or saline supplementation throughout the experimental period.

**Figure 1:**
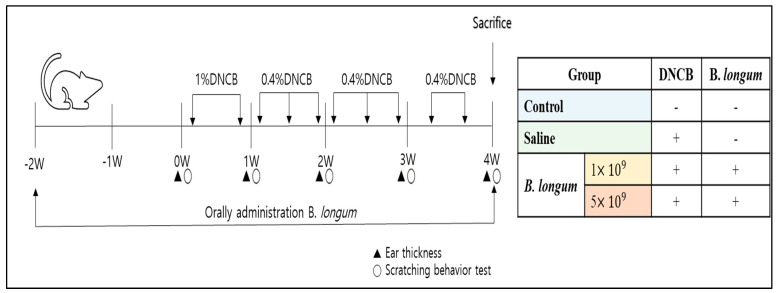
Experiment schedule for ACD induction by DNCB (2,4-Dinitrochlorobenzene) and daily supplementation with 1.0 mL saline solution or 1.0 × 10^9^ CFU and 5.0 × 10^9^ CFU *B. longum*. Control: mice were not induced by ACD treatment and did not receive *B. longum* or saline supplementation throughout the experimental period.

In this study, the concentration and dose of DNCB used for ACD induction and re-sensitization were similar to those reported by Son et al. [32], while the *B. longum* doses and administration method referred to those used in a previous study [33]. The ACD induction and *B. longum* administration schedule is briefly presented in Figure 1. On day 26, all the mice were sacrificed under CO_2_ conditions. 

### 2.5. Assays for the ACD 

#### 2.5.1. Body Weight and Symptoms Measurement

At 5-day intervals, the mice’ body weights were recorded. Ear thickness was measured weekly using a Digital micrometer (Mitutoyo Inc., Kawasaki, Japan). Scratching behavior was monitored once a week and counted (the number of pruritus instances) for 20 min by placing each mouse in a separate cage [34]. A single investigator was assigned to record these parameters throughout the experimental period to avoid artifacts.

#### 2.5.2. Histopathological Analysis

After scarification, the ear tissue samples were taken, fixed in 10% neutral-buffered formalin, and embedded in paraffin. The paraffin-embedded tissue with 5 μm thick sections was prepared using a cryostat microtome (HM525, Microm GmbH, Neuss, Germany). The sections (3 per mouse) were then stained with hematoxylin and eosin for epidermal thickness measurement. The other sections (3 per mouse) were stained with Toluidine blue for the detection of mast cells. All the stained samples were observed using an inverted microscope (Nikon Eclipse Ti, Nikon, Tokyo, Japan). Epidermal thickness and number of inflammatory mast cells were determined on 3 random fields of each sample.

### 2.6. Serum IgE Measurement

The level of IgE in the serum of all the mice at the end of the experiment was measured using an Enzyme-linked immunosorbent assay (ELISA). For this, whole blood samples were collected and centrifuged at 6000 rpm for 10 min at 25 °C. Thereafter, the serum IgE levels were measured using an ELISA kit according to the manufacturer’s instructions.

### 2.7. Western Blot Analysis

At the end of the experiment, the dorsal skin and left ear tissue samples were taken, homogenized, and lysed in a T-per tissue lysis buffer. Protein in the supernatant was collected after centrifuging the lysate at 13,000× rpm for 10 min at 4 °C. The tissue protein (4 mg/mL) was diluted using 4× Laemmli sample buffer and heated at 95 °C for 5 min. Each sample (20 µL) was loaded on 10% separating gel and 4% stacking gel at 110 volts. Following the electrophoresis, the protein in the gel was transferred onto a nitrocellulose membrane. The membranes with transferred proteins were washed with Tris-buffered saline-tween 20 (TBS-T) 3 times before blocking with 5% skim milk for 1 h at room temperature. The membranes reacted with primary antibodies, namely anti-GATA3 (GATA-protein binding 3) and anti-actin, at 4 °C overnight. After washing 3 times with TBS-T, the membranes were incubated with horseradish peroxidase-conjugated anti-mouse secondary antibodies for 1 h at room temperature. The antibody–protein reaction was detected using a maximum sensitivity substrate and observed with a Luminescent Image Analyzer System (Model: LAS-4000, Fujifilm, Tokyo, Japan). Density of Western blot bands was measured using ImageJ software (version 1.51j8) of the same system. Thereafter, GATA3/actin protein ratios were calculated by dividing the densitometric value of GATA3 by the densitometric value of actin. For standardization of band density, actin (a housekeeping protein) was used to ensure an equal protein loading across all the samples.

### 2.8. Real-Time Polymerase Chain Reaction (RT-PCR)

The mRNA expression of cytokine genes was measured using the RT-PCR technique. Total RNA was isolated from the dorsal and ear skin tissues using RNAiso plus reagent following the manufacturer’s instruction. The isolated RNA was converted into cDNA using prime Script 1st strand cDNA synthesis kit (Toyobo Com., Seoul, Republic of Korea) according to the manufacturer’s instruction. RT-PCR was carried out using GreenStar™ qPCR Master Mix (Bioneer Inc., Daejeon, Republic of Korea), cDNA (10 ng of total RNA equivalent), and primers specific to IL-4, IL-5, IL-13, IL-17, IFN-γ, TNF-α, and actin (reference gene), as listed in Table 1. The primers were designed and purchased from Bioneer Inc. (Daejeon, Republic of Korea). The RT-PCR was performed using a CFX96 instrument (Bio-Rad, Seoul, Republic of Korea) under conditions as follows: initial annealing temperature of 56 °C for 2 min, denatured at 95 °C for 3 min, followed by 40 cycles of denaturation and annealing at 95 °C/10 s and 72 °C/10 s, respectively. The expression level of the target genes was calculated using 2^−ΔCt^ method [35] and then normalized to actin mRNA level.

### 2.9. Statistical Analysis

Statistical analysis was carried out using a Statistical Analysis System (version 7.1; SAS Inst. Inc., Cary, NY, USA). Data were analyzed using ordinary one-way ANOVA. The ACD-induced groups with/without probiotic *B. longum* supplementation and the control were considered as the main effect, and the obtained results were considered random variables. Two analysis sets were carried out separately: one included the control and ACD-induced group without *B. longum* supplementation (only received saline solution), and the rest included the ACD-induced group with *B. longum* supplementation and the ACD-induced group without *B. longum* supplementation. Means were compared using Duncan’s Multiple Range Test. All values represent the mean ± standard deviation. Data are representative of three independent experiments. A value of *p* < 0.05 was used as the threshold for significance. 

## 3. Results and Discussion

### 3.1. Daily Supplementation with Probiotic B. longum Attenuates the ACD Symptom, Skin Barrier Damage, and Immune Responses

**Figure 2:**
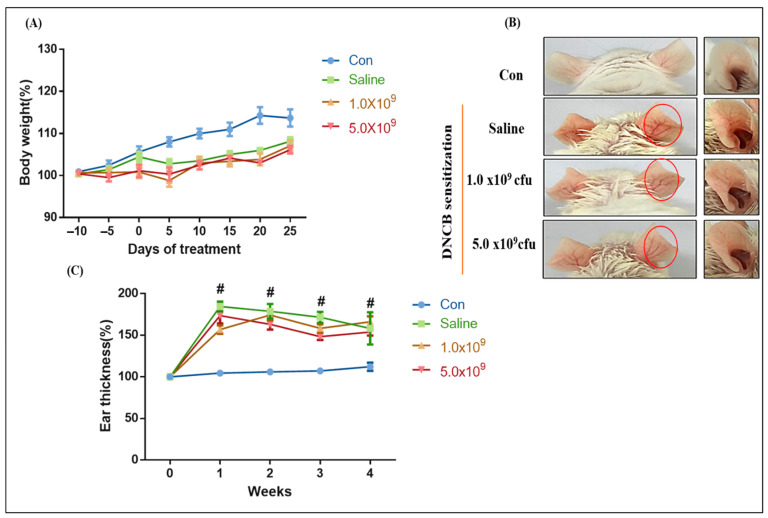
Symptoms of ACD-induced mice during the experimental period. Change in body weight (expressed in percentage, at 5 day-intervals) (**A**), ACD-like skin lesions (circles with red color indicate the erythematous and swollen signs with different severities depending on the groups) evaluated by macroscopic view (**B**). Ear thickness (% compared to initial measurement, day 0) measured by micrometer (**C**). Con (control): mice were not induced by ACD treatment, did not receive *B. longum* or saline supplementation throughout the experimental period; 1 × 10^9^ CFU or 5 × 10^9^ CFU *B. longum*: ACD-induced mice were daily supplemented with 1 × 10^9^ CFU or 5 × 10^9^ CFU *B. longum* during the experimental period; saline: ACD-induced mice daily received 1.0 mL saline solution during the experimental period. Results are expressed as means ± SD. (# *p* < 0.05 compared to the control group).

The change in body weight of the mice experimented on, recorded at 5-day intervals, is presented in Figure 2A. No difference in body weight occurred between the control and experimental groups (*p* > 0.05). However, it was observed that the control mice showed a slightly higher weight gain compared to the ACD-induced mice. This means that the ACD induction partly affected the growth rate of the animals. As shown in Figure 2B (red circle), the mice sensitized with DNCB showed the typical symptoms of ACD, such as ear edema, erythema, and swelling, compared to the control. However, these symptoms showed different levels depending on the groups: more severe in the ACD-induced group without *B. longum* supplementation (only received saline solution), and lesser in the groups with daily *B. longum* supplementation. This was further confirmed by a significantly higher ear thickness increment in the ACD-induced group without *B. longum* supplementation compared to the other remaining groups (*p* < 0.05). No significant differences in the ear thickness increment occurred between the control and ACD-induced groups with *B. longum* supplementation on all days examined. This signified the anti-inflammatory effect of probiotic *B. longum* in ACD conditions. In agreement with our results, Kim et al. [36] reported that administering probiotic *L. plantarum* LM1004 for 28 days significantly alleviated edema (indicated by a lower ear thickness increment) in mice with ACD. 

**Figure 3:**
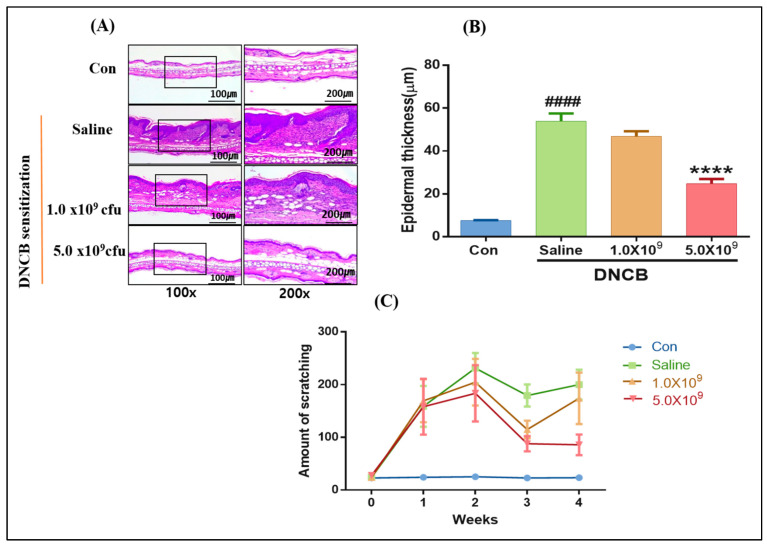
Histopathological results of ACD-induced mice who received 1.0 × 10^9^ CFU or 5.0 × 10^9^ CFU *B. longum* or 1.0 mL saline solution daily during the experimental period. Ear tissue sections stained with hematoxylin and eosin for epidermal thickness measurement were observed under an inverted microscope at 100× and 200× magnification (**A**). Epidermal thickness (µm) averaged from fifteen fields (**B**), scratching behavior measured every week for 20 min (**C**). Con (control): mice were not induced by ACD treatment and did not receive *B. longum* or saline supplementation throughout the experimental period. Results are expressed as means ± SD. (#### *p* < 0.0001, compared to the control group; **** *p* < 0.0001, compared to the ACD-induced group daily received saline solution).

According to our histopathological analysis result (Figure 3), the ACD-induced mice without *B. longum* supplementation showed the highest epidermal thickness (58.05 ± 0.16 µm) compared to the control group (Figure 3A,B) (*p* < 0.0001). Meanwhile, the daily supplementation with *B. longum*, especially at a higher dose (5 × 109 CFU), significantly reduced the epidermal thickness (*p* < 0.0001). Researchers have proposed that an increase in cytokines production by T-helper 2 (Th2) cells, in turn, triggers the Th2 and antigen-presenting cells interaction, leading to a deficiency of filaggrin protein and resulting in cell death, inflammation, and skin-barrier damage [37,38]. Scratching behavior is considered the typical symptom of ACD. In the present study, the number of scratching appearances was monitored and counted once per week for 20 min for both the control and ACD-induced mice. Figure 3C shows no scratching behavior was observed for the control group. Although no significant difference in the number of scratching times occurred among the ACD-induced mice, the group without *B. longum* supplementation (saline solution received daily) had more frequent scratching behaviors. Itching or scratching is caused by the action and binding of histamine released from mast cells on the H1 receptor on the endothelial cells [39]. 

**Figure 4:**
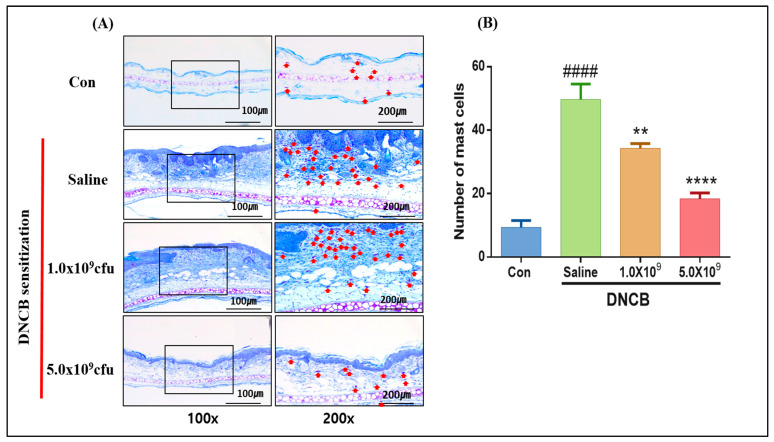
Histopathological result of ACD-induced mice who received 1.0 × 10^9^ CFU or 5.0 × 10^9^ CFU *B. longum* or 1.0 mL saline solution daily during the experimental period. Ear tissue sections were stained with Toluidine blue for mast cell counting at 100× and 200× magnification. Red arrows indicate the mast cells (**A**) and several mast cells counted at random fields (**B**). Con (control): mice were not induced by ACD treatment and did not receive *B. longum* or saline supplementation throughout the experimental period. Results are expressed as means ± SD. (#### *p* < 0.0001 compared to the control group; ** *p* < 0.01, **** *p* < 0.0001 compared to the ACD-induced group that received saline solution daily).

Mast cells are known as the immune cells, which exert a central role in the inflammatory and immediate allergic reactions as they are responsible for producing potent inflammatory mediators such as cytokines and proteinases [40]. Many mechanisms, such as chemical substances, endogenous mediators, and IgE molecules, have contributed to mast cells’ proliferation and differentiation in the adaptive immune response [41]. Mast cells have been shown to stain blue with some dyes, such as Toluidine, by heparin in their cytoplasm granules [42]. In the present study, to examine whether ACD induction causes an increase in the recruitment of the mast cells, the ear tissue samples were stained with Toluidine, and the results are shown in Figure 4. The number of mast cells in the ACD-induced mice without *B. longum* supplementation was higher compared to the control (*p* < 0.0001). As expected, the daily supplementation with *B. longum* at both doses significantly reduced the number of mast cells. It was also observed that the higher the supplemented *B. longum* dose, the lower the mast cell number (Figure 4B). In line with our result, Kwon et al. [43] reported that daily administration with a probiotic mixture containing *L. leuteri, casei, acidophilus,* and *B. bifidium* (5 × 10^8^ CFU each) significantly reduced the immune cell populations in allergic disease-induced mice. In a study conducted by Kim et al. [44], mice were induced with food allergy by ovalbumin (an allergen) and then supplemented daily with the same dose of *B. longum* KACC91563. The allergy symptoms and number of mast cells were significantly reduced. Studies have found that once activated, the mast cells quickly migrate to inflammation sites (e.g., skin), releasing cytokines and histamine to attract the immune defense system-participating players [41].

**Figure 5:**
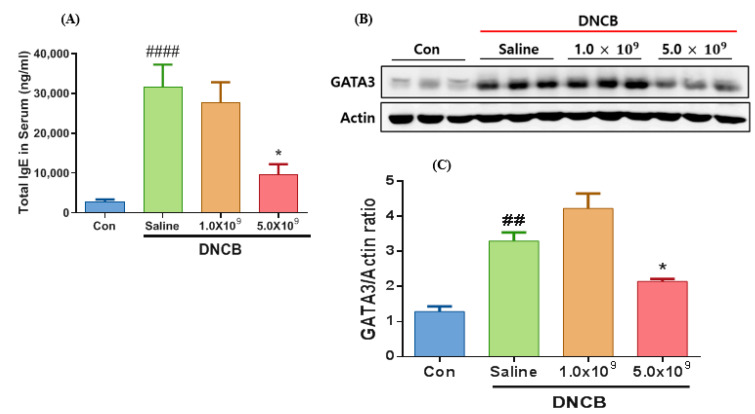
Humoral immune response and GATA3 protein expression in ACD-induced mice daily received 1.0 × 10^9^ CFU or 5.0 × 10^9^ CFU *B. longum* or 1.0 mL saline solution during the experimental period—total IgE level in serum measured with ELISA kit (**A**). The protein band of GATA3 (MW: 50 kDa) and actin (MW: 42 kDa) in the mouse ear tissues was detected by Western blotting (**B**) and its protein intensity about actin (**C**). Con (control): mice were not induced by ACD treatment and did not receive *B. longum* or saline supplementation throughout the experimental period. Results are expressed as means ± SD. (## *p* < 0.01, #### *p* < 0.0001 compared to the control group; * *p* < 0.05 compared to the ACD-induced group that received saline solution daily).

ACD is characterized by its delayed-type hypersensitivity (development of at least two days or longer is required) or IgE-mediated hypersensitivity reaction type [45]. During the allergic immune response, the secreted IgE level (millions of IgE molecules) will cover the mast cell’s surface at the IgE receptors, stimulating the release of cytokines and histamines [46]. After scarification, the serum samples were collected and measured for this antibody type to examine whether the ACD induction by DNCB leads to an increase in the IgE level. It was observed that the ACD-induced mice without *B. longum* administration (only received saline solution) had the highest IgE level compared to the control (Figure 5A) (*p* < 0.0001). However, the ACD-induced groups with daily *B. longum* supplementation (e.g., at a dose of 5.0 × 10^9^ CFU) exhibited a significantly lower IgE level (*p* < 0.05) compared to the ACD-induced group without *B. longum* supplementation. Our result aligns with Kim et al. [36], who showed that the oral administration of *L. plantarum* LM1004 significantly reduced the total serum IgG level in ACD-induced mice. In a study conducted by Li et al. [47], mice were sensitized with 2,4-dinitrofluorobenzene (DNFB) to induce ACD and then treated with IL-37 (anti-inflammatory cytokine), and a lower IgE level was found compared to the ACD-induced group without IL-37 treatment. These authors also found a positive correlation between the IgE and mast cell number. Recently, the neutralization of IgE has been proposed as a promising therapy for allergic diseases. An et al. [48] have found that neutralization of IgG (e.g., utilization of anti-IgE antibody) combined with *B. longum* KACC91563 supplementation efficiently reduced the allergic responses in food allergy-induced mice.

GATA3 binding protein 3, named based on its ability to reorganize the G-A-T-A nucleotides sequence in its target gene, is expressed by both the Th1 and Th2 cells [49]. GATA3 is an indispensable transcription factor for the proliferation, differentiation, and cytokine production induction of Th2 cells [50]. Our results showed that all the control and experiment groups showed the GATA3 expression (Figure 5B). However, GATA3 protein expression elevated (by 3.5 times) in the ACD-induced mice without *B. longum* supplementation in comparison with the control (*p* < 0.01) (Figure 5C). Notably, the ACD-induced mice supplemented with the probiotic *B. longum* (e.g., 5.0 × 10^9^ CFU) exhibited a significantly (*p* < 0.05) lower GATA3 protein expression compared to the ACD-induced mice without *B. longum* treatment (Figure 5C). This signifies that the daily supplementation with 5.0 × 10^9^ *B. longum* efficiently abated the GATA3 protein expression, possibly contributing to the ACD alleviation. Supporting the present findings: Tamauchi et al. [51] detected GATA-3 expression in ear skin tissue of ACD-induced mice. Wu et al. [52] reported an increase in GATA3 expression level in ACD-induced mice by CNFB and re-sensitized on the right ear, compared with control (non-ACD induction). Similarly, Tiwari et al. [53] used an allergic disease-induced murine model and found that abating GATA3 expression alleviates disease progression. 

### 3.2. Daily Supplementation with Probiotic B. longum Suppresses the Inflammatory Cytokine Production

**Figure 6:**
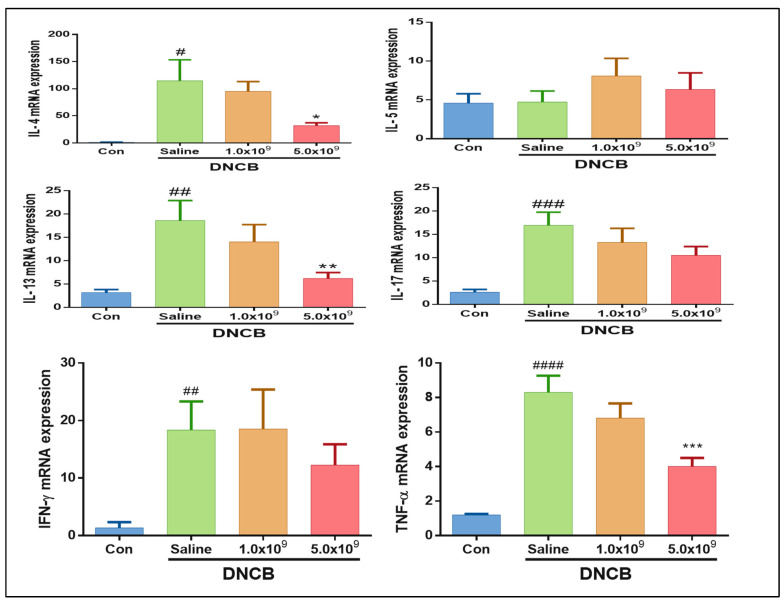
mRNA expression of T-cell related cytokines (IL-4, IL-5, IL-13, IL-17, IFN-gamma, and TNF-alpha) in ear tissues of ACD-induced mice daily received 1.0 × 10^9^ CFU or 5.0 × 10^9^ CFU *B. longum* or 1.0 mL saline solution during the experimental period. Con (control): mice were not induced by ACD treatment and did not receive *B. longum* or saline supplementation throughout the experimental period. Results are expressed as means ± SD (# *p* < 0.05 ## *p* < 0.01, ### *p* < 0.001, #### *p* < 0.0001 compared to the control; * *p* < 0.05, ** *p* < 0.01, *** *p* < 0.001 compared to the ACD-induced group daily received saline solution).

Cytokines are known to be small proteins that are indispensable for inflammation and immune response modulation, and they are subdivided according to their source of production (such as cell types) as well as their type of immune response (innate or adaptive immune response) [54]. In the present study, to elucidate the immune responses by related cell populations in the ACD-induced mice, the level of cytokines mRNA expression, including IL-4, IL-5, IL-13, IL-17, IFN-γ, and TNF-α, was measured. As shown in Figure 6, a higher mRNA expression level of IL-4 (*p* < 0.05), IL-13 (*p* < 0.01), and IL-17 (*p* < 0.01) was observed in the ACD-induced mice without *B. longum* supplementation (only received saline solution) in comparison to the control. At the same time, the ACD-induced group that received 5 × 10^9^ CFU *B. longum* daily had a lower IL-4 (*p* < 0.05) and IL-13 (*p* < 0.01) mRNA expression level in comparison with the ACD-induced mice without *B. longum* supplementation. IL-4, IL-5, and IL-13 are mainly produced by Th2 cells and other cell types such as mast cells, dendritic cells, T cells, and eosinophils [13,54]. IL-4 plays a multifunctional role in the adaptive immune response by stimulating B cell proliferation and antibody (IgE) production [55]. Regarding IL-5 and IL-13, the role (e.g., stimulating eosinophils proliferation and antibody production by activated B cells) in the immune responses has been reported [56]. Furthermore, they both can drive inflammatory responses in allergic diseases [57]. 

Similarly, the IFN-γ and TNF-α mRNA expression levels were significantly lower in the ACD-induced mice without *B. longum* supplementation (only received saline solution). A suppressive effect of *B. longum* supplementation on the TNF- α mRNA expression was observed. However, this effect (*p* < 0.001) was only observed in the ACD-induced mice supplemented daily with a higher *B. longum* (5.0 × 10^9^ CFU) dose. The daily supplementation of *B. longum* at both doses showed a decreasing trend of IFN-γ mRNA expression in the ACD-induced mice but not significantly different (*p* > 0.05). Similar to our results, researchers have also reported a suppressive effect of probiotic supplementation on cytokine production in the ACD-induced mouse model. For instance, Kwon et al. [58] used DNCB to induce mice with ACD and then treated them with L. sakei, and they reported a decrease in Th2-derived cytokine expression. Similarly, Chapat et al. [21] induced mice with ACD by DNFB, then treated with *L. casei,* and found a decrease in IFN-γ level. Thus, the daily supplementation with 5.0 × 10^9^ *B. longum* effectively suppresses these cytokines’ production from the immune cells.

Overall, in the present study, the daily supplementation with *B. longum* alleviated the DNCB-induced ACD symptoms, such as reduced ear swelling, epidermal damage, immune cell recruitment (reduced mast cell number), IgE production, and cytokines expression. The possible mechanisms underlying these phenomena could be attributed to the contribution of supplemented probiotic *B. longum* to maintaining a balanced gut microbiota population, which might modulate the allergic immune responses in the ACD condition. Scientific studies have shown an immunological crosstalk between gut and skin in which the gut epithelial cell barrier, mucus layer, IgA, and immune cells (T cells and dendritic cells) all contribute to mucosal firewall enhancement, resulting in prevention of gut and skin from inflammation [59]. Furthermore, gut microbial communities play a vital role in maintaining overall homeostasis of skin and gut barrier integrity as they can convert indigestible complex polysaccharides into vitamins and short-chain fatty acids, which are important immune response modulators [14,60]. In the present study, the number of *B. longum* in the feces samples of ACD-induced mice after supplementation was also found at around 5–6 log CFU/g. In a survey by Sasajima et al. [22], DNFB was used to induce mice with ACD and daily supplemented with 10^6^ CFU *B. pseudolongum*, and these authors found a high living count (5–7 log CFU/g) in feces samples. These authors proposed that the proliferation of BB in the gastrointestinal tract could be mainly responsible for the alleviation of ACD. 

## 4. Conclusions

ACD is known as a chronic inflammatory skin disease occurring at all ages with severe symptoms that negatively impact our psychological condition and quality of life. In the present work, the ACD was induced by DNCB allergen through an animal model, and the therapeutic effect of probiotic *B. longum* was investigated. The inflamed sites’ serum and skin tissue samples were collected and analyzed. Before 2 weeks of ACD induction and during the re-sensitizing period, the daily supplementation with probiotic *B. longum* significantly alleviated symptoms of disease severity (e.g., ear swelling and epidermal damage) and immune response (e.g., reduced immune cell recruitment, serum IgE level, and cytokines expression). When comparing the therapeutic efficiency between the two doses of probiotic *B. longum*, a better result was observed for the higher dose (5 × 10^9^ CFU). Based on the results obtained from this animal model study, it may be said that daily supplementation with 5.0 × 10^9^ CFU probiotic *B. longum* could be an effective method for preventing and treating inflammatory skin diseases caused by direct contact with exogenous reactive allergens. Further study is needed to investigate the alleviative effect of *B. longum* KACC91563 against the ACD in animal models and patients via foods or dietary supplements.

## Figures and Tables

**Table 1:** Primer sequences used for amplification of interested genes using RT- PCR.

Gene	Accession Number	Forward	Reverse
Actin	NM_007393.5	5′-GAAATCGTGCGTGACATCAAAG-3′	5′-TGTAGTTTCATGGATGCCACAG-3′
Interleukin 4 (IL-4)	NM_021283.2	5′-TCACTGACGGACCAGAGCTA-3′	5′- TGTGAGGACGTTTGGCACAT-3′
Interleukin 5 (IL-5)	NM_010558.1	5′-CGTGGGGGTACTGTGGGAAAT-3′	5′-AATCCAGGAACTGCCTCGTC-3′
Interleukin 13 (IL-13)	NM_008355.3	5′-TGCCATCTACAGGACCCAGA-3′	5′-CTCATTAGAAGGGGCCGTGG-3′
Interleukin 17 (IL-17)	NM_010552.3	F 5′-ACTACCTCAACCGTTCCACCT-3′	5′-TTCCCTCCGCATTGACACACT-3′
Interferon γ (IFN-γ)	NM_008337.4	5′-GATGCATTCATGAGTATTGCCAAGT-3′	5′-GTGGACCACTCGGATGAGCTC-3′
Tumor necrosis factor α (TNF-α)	NM_013693.3	F 5′-GAACTGGCAGAAGAGGCACT-3′	5′-AGGGTCTGGGCCATAGAACT-3′

## Data Availability

The original contributions presented in the study are included in the article, further inquiries can be directed to the corresponding author.
